# The Effects of Passenger Risk Perception During the COVID-19 Pandemic on Airline Industry: Evidence From the United States Stock Market

**DOI:** 10.3389/fpsyg.2021.795940

**Published:** 2022-01-04

**Authors:** Zhou Lu, Linchuang Zhu, Zhenhui Li, Xueping Liang, Yuan Zhang

**Affiliations:** ^1^School of Economics, Tianjin University of Commerce, Tianjin, China; ^2^School of Economics and Management, Communication University of China, Beijing, China

**Keywords:** risk perception, COVID-19, Quandt–Andrews test, risk aversion, airline industry

## Abstract

The COVID-19 pandemic has caused a dramatic reshaping of passenger risk perception for airline industry. The sharp increase in risk aversion by air passenger has caused a disastrous impact on the tourism service industry, particularly airline industry. Although the existing literature has provided a lot of studies on the impact of the pandemic on travel industry, there are very few studies discussing the impact of change in passenger risk perception on the stock market performance of airline industry. This study considers two types of airline companies, full-service and low-cost. In order to overcome the traditional problem of the Chow test, Quandt–Andrews test is used to identify structural change points during the pandemic in the stock prices of United States airline companies. The result shows that an industry-wide structural change in the stock market performance indeed is found to take place during the pandemic for United States airline companies. Meanwhile, no significant difference is found in the structural change date between the two types of airline companies. The selected airline companies are found to be clustered toward the end of 2020 (November and December) in their structural change dates. Although the strike of the COVID-19 pandemic on airline industry has proven to be widespread and profound, our investigation implies that air passengers have gradually adapted to the new normal of travel activities at some level and partly rebuild their sense of safety under the strict epidemic-control measures.

## Introduction

In 2020, the COVID-19 pandemic has spread widely throughout the United States. As of October 5, 2021, there have been more than 44 million confirmed cases in the United States, and the cumulative death toll has exceeded 720,000. There are more than 200 countries or regions around the world with confirmed COVID-19 cases As of October 5, 2021, the cumulative number of confirmed diagnoses worldwide has exceeded 236 million, and the cumulative death toll has exceeded 4.82 million, which will undoubtedly have a huge impact on the global economy. The COVID-19 pandemic has caused the stagnation of social and economic activities and caused panic and large fluctuations in the global financial market. In order to combat the epidemic, many countries have adopted measures such as border-closing and travel restriction. Epidemic prevention measures include social distancing and travel minimization. The COVID-19 epidemic has also had a disastrous impact on the tourism service industry. The United Nations World Tourism Organization (UNWTO) report shows that the number of global international tourists in 2020 has declined by more than 1 billion, up to 74% of 2019 number. And global tourism revenue has decreased 1.3 trillion United States dollars, a decrease of 11 times that of the 2009 global financial crisis, with a direct loss of 100 million to 120 million jobs (UN-WTO, 2020).

During the epidemic, governments have demanded travel minimization. This has a devastating impact on the global airline industry: passenger traffic was 1.8 billion in 2020, a decrease of 60.2% from the 4.5 billion in 2019. Year 2020 witnessed the largest fall in air passenger traffic since 1950 (International Air Transport Association, 2020). One of the major drivers of this substantial decline in airline passenger travel is the risk perception of air passengers. This study aims to investigate the effect of the considerable change in passenger risk perception on airline industry by looking at stock market performance. Particularly, the research question to address is whether there exists an identifiable change in the stock market performance of airline industry. In this paper, sample United States airline companies were selected to represent different airline types. The Quandt–Andrews test was used to determine structural change points so as to better investigate the effect of passenger risk perception in the context of COVID-19. The rest of the paper is structured as follows. The Literature Review section examines the relevant existing literature. The Data and Methodology section discusses data selection and empirical model. The Empirical Results section lays out the results and the Conclusion section discusses policy implications.

## Literature Review

The existing literature on the COVID-19 pandemic and airline industry is concentrated on the economic impact of the pandemic on airline industry. Maneenop and Kotcharin (2020) examined the short-term effect of COVID-19 outbreak on 52 listed airline companies’ stock prices. Based on their results, airline stock return has significantly declined after three major COVID-19 announcements were made. Based on global air passenger traffic data, Lacus et al. (2020) studies the impact of the travel ban on airline industry. Their study found that the impact caused an average decline in global GDP by 0.02–0.12% in the first quarter of 2020. As for the COVID-19 related risk of air travel, Zhang et al. (2020) constructed a risk index based on daily dynamic international aviation data to measure the imported case risk on inbound international flights. They found that after China imposed restrictions on inbound flights, the imported case risk could be effectively controlled and the number of imported cases has fallen by about 50%.

In terms of business models, airline companies can be divided into two types, low-cost versus full-service. This distinction becomes a common practice in relevant empirical studies. Gong et al. (2006) suggests that it is better to distinguish between different types of airlines for empirical investigation. Low cost airlines are mainly characterized by online ticket booking, no free meals, standardized flight fleet, high aircraft utilization, and service outsourcing (Mason and Morrison, 2009; Teece, 2010; Diaconu, 2012; Pereira and Caetano, 2015; Dobruszkes and Wang, 2019). In terms of routes, due to strict regulations on long-distance transportation services, low-cost airlines mainly serve short- and medium-distance routes (Jiang, 2013; Albers et al., 2020). By contrast, the most distinctive feature of full service airlines is customer segmentation, the provision of a variety of different cabins (from first-class cabins, business class, to economy class). In addition, full-service airlines also have the following characteristics: in-flight meals, personalized services, large-scale network routes, extensive distribution channels, and complete supporting facilities (O’Connell, 2019).

Some scholars explore how different types of airline companies were affected by major travel crises. Ding et al. (2021) evaluate the connection between corporate characteristic and stock returns. They found that the decline in stock prices is more significant for firms with stronger financial conditions (more cash, less debts, and larger profits) and with less exposure to global supply chains. Carter and Simkins (2004) investigate the reaction of airline stock prices to 9/11 terrorist attack and found that there exists a variance in the impact on airline stock prices. Flouris and Walker (2005) also explore the effects of 9/11 terrorist attack on low-cost airline and full-service airline. The results found that the performance of Southwest Airlines, a typical low-cost airline, was much better than full-service airlines. Kökény et al. (2021) examine the impact of COVID-19 on the stock market performance of listed European airlines. In addition, the existing studies indicate that the impact of an external shock varies across different airline companies.

There has been abundant existing literature on the impact of epidemic on airline industry. However, there are few studies discussing the heterogeneous impact of public health events such as the COVID-19 epidemic on different types of airline industry. Therefore, this paper carefully distinguishes between different airline types in studying the impact of passenger risk perception driven by the COVID-19 epidemic on airlines. The Quandt–Andrews test is used to identify structural change points in the stock prices of United States airline industry. Then, based on the results from the empirical analysis of airline industry, a comparative analysis is conducted on the different types of airlines in the United States.

## Data and Methodology

We use the time series data on stock price from Wind-Economic Database for this study. For the selection of sample period, it should be noted that the COVID-19 pandemic did not strike the United States economy at large scale until March, 2020. The number of confirmed cases before March stayed below 100. By contrast, the number of confirmed cases began to experience exponential growth in March 2020. We consider the period of February 28, 2020 to September 14, 2021, which contains a total of 564 trading days. Five airline companies (American Airlines, Alaska Airlines, Delta Air Lines, United Airlines, and Hawaiian Airlines) were selected as sample full-service airlines. Meanwhile, Southwest Airlines, JetBlue Airways, Spirit Airlines, Allegiance Airlines, Ryanair were selected as sample low-cost airlines. Instead of closing stock quotations and opening stock prices, we use the average daily stock price for selected United States airline companies.

The Chow test (Chow, 1960) has been widely used for parameter stability testing in the existing literature. This method is very effective in testing structural change in economic indicators at a given time. However, the Chow test has limitations as this test requires that the tested model does not miss important variables and there is no systematic error in the model setting. It also assumes that the explanatory variables are not correlated with the random disturbance for each sub-interval. Given the limitations of the Chow test, Quandt, and Andrews proposed a test method for unknown change points. The statistics of Quandt–Andrews test include three types: (1) the largest statistic, obtained from the Chow test; (2) Ave statistic, the simple arithmetic mean of *F*-statistic values obtained from the Chow test; and (3) Exp statistic. The result of the test output includes these three statistic *F*-statistic and LR statistic. From each single Chow test, two constrained statistics can be obtained: likelihood ratio *F*-statistic and Wald *F*-statistics. The likelihood ratio *F*-statistic is a statistic based on the comparison of the residual sum of squares between the constrained and the unconstrained models. And the Wald *F*-statistic is calculated according to the standard Wald test. In an ordinary linear model, these two statistics turn out to be equal to each other. A single Chow test for split point can be summarized into three different statistics: the maximum statistics, the Exp statistic, and the Ave statistic (Andrews, 1993; Andrews and Ploberger, 1994).

The maximum statistic is the statistic obtained by taking the maximum value of a single statistic obtained in the test:


(1) $$\max F=\max_{\tau_{1}\leq \tau \leq \tau_{2}}F\left(\tau \right)$$

The Exp statistics is obtained as follows:


(2) $$\exp F=\ln\left(\frac{1}{k}\sum_{\tau =\tau_{1}}^{\tau_{2}}\exp\left(\frac{1}{2}F\left(\tau \right)\right)\right)$$

The Ave statistic is the simple average of the individual statistics obtained:


(3) $$AveF=\frac{1}{k}\sum_{\tau =\tau_{1}}^{\tau_{2}}F\left(\tau \right)$$

It should be noted that the distribution is non-standard for the above three statistics. Andrews (1993) derived the true distribution and then Hansen (1998) gives asymptotical *p*-value for the three statistics.

## Empirical Results

The Quandt–Andrews test is conducted on the above mentioned stock price time series data to test the parameter stability and to identify structural change. Then, the above three statistics are calculated and the asymptotic *p*-value is obtained for each calculated statistic.

Table 1 shows the results of the Quandt–Andrews test on the selected full-service airlines and low-cost airlines from the United States airline industry. Since the likelihood ratio *F*-statistic and Wald *F*-statistic are the same in an ordinary linear model, only the Wald *F*-statistic is shown in Table 1. The three major statistics all accept the hypothesis that there exist structural change points in the time series data at a significance level of 1%. As shown in Table 1, the distribution of structural change dates is relatively concentrated. The structural change for all representative companies with the exception of American Airlines is show to take place in the last 2 months of 2020 (November 2020 through December 2020). Meanwhile, no significant difference is found in structural change dates between low-cost airlines and full-service airlines. This result shows that there is no disparity in the pandemic impact across different airline types.

**TABLE 1:** Quandt–Andrews test results.

Business model	Airline company	Structural change	Statistics		*p*-Value
Full-service	American Airlines	January 28, 2021	Maximum Wald *F*-statistic	1213.413	0.000
			Exp Wald *F*-statistic	601.2568	0.000
			Ave Wald *F*-statistic	437.8184	0.000
	Alaska Air	November 23, 2020	Maximum Wald *F*-statistic	1247.887	0.000
			Exp Wald *F*-statistic	618.749	0.000
			Ave Wald *F*-statistic	529.046	0.000
	Delta	November 16, 2020	Maximum Wald *F*-statistic	872.7903	0.000
			Exp Wald *F*-statistic	431.4397	0.000
			Ave Wald *F*-statistic	342.5232	0.000
	United Airlines	November 24, 2020	Maximum Wald *F*-statistic	577.2857	0.000
			Exp Wald *F*-statistic	283.4525	0.000
			Ave Wald *F*-statistic	275.0632	0.000
	Hawaiian Airlines	November 09, 2020	Maximum Wald *F*-statistic	877.48	0.000
			Exp Wald *F*-statistic	433.2051	0.000
			Ave Wald *F*-statistic	361.0949	0.000
Low-cost	Southwest Airlines	November 16, 2020	Maximum Wald *F*-statistic	950.1833	0.000
			Exp Wald *F*-statistic	469.8046	0.000
			Ave Wald *F*-statistic	455.259	0.000
	JetBlue	November 09, 2020	Maximum Wald *F*-statistic	758.9487	0.000
			Exp Wald *F*-statistic	373.9351	0.000
			Ave Wald *F*-statistic	357.6673	0.000
	Spirit Airlines	December 03, 2020	Maximum Wald *F*-statistic	912.8687	0.000
			Exp Wald *F*-statistic	451.1336	0.000
			Ave Wald *F*-statistic	379.2932	0.000
	Allegiant Air	December 03, 2020	Maximum Wald *F*-statistic	1278.903	0.000
			Exp Wald *F*-statistic	634.2814	0.000
			Ave Wald *F*-statistic	525.5729	0.000
	Ryanair	November 09, 2020	Maximum Wald *F*-statistic	1853.522	0.000
			Exp Wald *F*-statistic	921.2082	0.000
			Ave Wald *F*-statistic	637.6137	0.000

According to the structural change dates shown in Table 1, a comparative analysis was conducted on the descriptive statistics of the stock prices. The results are shown in Table 2. We find that pre-structural-change mean stock prices are shown to be higher than the post ones for all airline companies, which reflects a noticeable drop in passenger risk perception. Two low-cost carriers, Spirit Airlines and Allegiant Air, saw the biggest gains of more than 80%. Generally, the percentage change in the mean stock price is greater for low-cost airlines than for full-service airlines. Ryanair shows a significant decrease from 11.38 to 4.64 in price vitality indicating that its stock price became more stable after the structural change.

**TABLE 2:** Descriptive statistics of stock prices before and after structural change unit: USD.

	Variable	Before the structural			After the structural			Percentage change
		change point			change point			
		Obs	Mean	SD	Obs	Mean	SD	
Full-service	American Airlines	219	13.35	2.33	150	21.38	1.92	60.1
	Alaska Air	175	36.42	5.77	194	60.36	7.09	65.7
	Delta	170	29.21	5.51	199	43.29	3.57	48.2
	United Airlines	176	34.41	7.01	193	50.11	5.51	45.6
	Hawaiian Airlines	165	13.63	2.21	204	22.62	3.36	66.0
Low-cost	Southwest Airlines	170	35.73	4.78	199	53.39	6.02	49.4
	JetBlue	165	10.96	1.75	204	16.97	2.32	54.8
	Spirit Airlines	182	16.63	3.79	187	30.76	5.08	84.9
	Allegiant Air	182	114.91	25.63	187	208.92	26.63	81.8
	Ryanair	165	71.36	11.38	204	109.02	4.64	52.8

The outbreak of the COVID-19 early 2020 had exerted a disastrous impact on the United States airline industry regardless the airline type. This impact was seen in the substantial fall in airline stocks. However, the negative impact of the COVID-19 outbreak gradually diminished as air passenger risk perception reduced substantially. There are mainly two drivers for this risk perception reduction. First, to control in-flight transmission, United States airline industry has taken various preventive measures including mask use, temperature screening, and boarding requirement of negative testing results. Secondly, as air passengers have adapted to the new travel conditions, air travel demand, which was depressed during the outbreak of COVID-19 outbreak, experienced a moderate level of recovery.

## Conclusion

This study analyzes the high-frequency daily stock market data to investigate the impact of a change in passenger risk perception driven by the COVID-19 epidemic on airline industry. We consider the heterogeneous impact of passenger risk perception on different airline types by examining the average daily stock price of the United States airline companies selected for full-service airlines and for low-cost airlines.

The result shows that there indeed exists an industry-wide structural change in stock market performance taking place during the pandemic for United States airline companies and the selected United States airline companies are shown to be clustered toward the end of 2020, particularly November and December, in stock price. Meanwhile, no significant difference is found in the structural change date between the two types of airline companies. We find that pre-structural-change mean stock prices are shown to be higher than the post ones for all airline companies.

Although global public health emergency can exert devastating impact on airline industry air, our results imply that passengers adapt themselves gradually to the new normal of travel activities amid potential lockdown and flight cancellation. Meanwhile, air passengers might respond to epidemic-control measures positively and update their perception of travel risk continuously. This behavior pattern is reflected in the structural change of airline stock prices that is found to take place toward the end of 2020. In addition, this implication of steady reduction in passenger risk perception seems to be consistent with some findings in the existing literature (Zhang et al., 2020).

Regarding policy implications, our results imply that the airline industry regulators should give more importance to the effect of COVID-19 prevention measures. Although the chance of in-flight transmission turns out to be very low, strict preventive measures including mask use, distance seating, temperature screening, and pre-boarding testing all help air passengers rebuild their sense of safety and reduce their risk perception. In the face of major public health events such as the COVID-19 epidemic, airline industry regulators should use big data in monitoring the trend and formulating policy responses in a timely manner. Meanwhile, quarantine of travelers may delay transmission or re-transmission of the virus, but it substantially increases the cost of long-haul travel particularly. By contrast, free cancellation, flexible bookings, and travel warning updates might help travelers handle COVID-19 related uncertainties. For future study, the question of how the measures that increase air travel flexibility affect air passenger risk perception might be worth investigation.

## Data Availability Statement

The original contributions presented in the study are included in the article/supplementary material, further inquiries can be directed to the corresponding author/s.

## Author Contributions

ZL: conceptualization, methodology, formal analysis, writing–revision, and project management. LZ: methodology, data processing, and writing–original draft preparation. ZHL: conceptualization and funding acquisition. XL: methodology, writing–original draft preparation, and funding acquisition. YZ: methodology, data processing, and writing–revision draft preparation. All authors contributed to the article and approved the submitted version.

## Conflict of Interest

The authors declare that the research was conducted in the absence of any commercial or financial relationships that could be construed as a potential conflict of interest.

## Publisher’s Note

All claims expressed in this article are solely those of the authors and do not necessarily represent those of their affiliated organizations, or those of the publisher, the editors and the reviewers. Any product that may be evaluated in this article, or claim that may be made by its manufacturer, is not guaranteed or endorsed by the publisher.
